# The *Trypanosoma cruzi* Virulence Factor Oligopeptidase B (OPBTc) Assembles into an Active and Stable Dimer

**DOI:** 10.1371/journal.pone.0030431

**Published:** 2012-01-19

**Authors:** Flávia Nader Motta, Izabela M. D. Bastos, Eric Faudry, Christine Ebel, Meire M. Lima, David Neves, Michel Ragno, João Alexandre R. G. Barbosa, Sônia Maria de Freitas, Jaime Martins Santana

**Affiliations:** 1 Pathogen-Host Interface Laboratory, Department of Cell Biology, The University of Brasília, Brasília, Brazil; 2 Faculty of Ceilândia, The University of Brasília, Brasília, Brazil; 3 INSERM, UMR-S 1036, Biology of Cancer and Infection, Grenoble, France; 4 CNRS, ERL 5261, Bacterial Pathogenesis and Cellular Responses, Grenoble, France; 5 UJF-Grenoble 1, Biology of Cancer and Infection, Grenoble, France; 6 CEA, DSV/iRTSV, Biology of Cancer and Infection, Grenoble, France; 7 CEA, Institut de Biologie Structurale Jean-Pierre Ebel, Grenoble, France; 8 CNRS, Institut de Biologie Structurale Jean-Pierre Ebel, Grenoble, France; 9 Université Joseph Fourier – Grenoble 1, Institut de Biologie Structurale Jean-Pierre Ebel, Grenoble, France; 10 Programa de Pós-Graduação em Ciências Genômicas e Biotecnologia, Universidade Católica de Brasília, Brasília, Brazil; 11 Laboratory of Biophysics, Department of Cell Biology, The University of Brasília, Brasília, Brazil; State University of Campinas, Brazil

## Abstract

Oligopeptidase B, a processing enzyme of the prolyl oligopeptidase family, is considered as an important virulence factor in trypanosomiasis. *Trypanosoma cruzi* oligopeptidase B (OPBTc) is involved in host cell invasion by generating a Ca^2+^-agonist necessary for recruitment and fusion of host lysosomes at the site of parasite attachment. The underlying mechanism remains unknown and further structural and functional characterization of OPBTc may help clarify its physiological function and lead to the development of new therapeutic molecules to treat Chagas disease. In the present work, size exclusion chromatography and analytical ultracentrifugation experiments demonstrate that OPBTc is a dimer in solution, an association salt and pH-resistant and independent of intermolecular disulfide bonds. The enzyme retains its dimeric structure and is fully active up to 42°C. OPBTc is inactivated and its tertiary, but not secondary, structure is disrupted at higher temperatures, as monitored by circular dichroism and fluorescence spectroscopy. It has a highly stable secondary structure over a broad range of pH, undergoes subtle tertiary structure changes at low pH and is less stable under moderate ionic strength conditions. These results bring new insights into the structural properties of OPBTc, contributing to future studies on the rational design of OPBTc inhibitors as a promising strategy for Chagas disease chemotherapy.

## Introduction

Chagas disease (American Trypanosomiasis) is a multisystemic illness resulting from the infection with the intracellular protozoan parasite *Trypanosoma cruzi*, which is able to invade a wide variety of mammalian cells. It affects millions of people in Latin America and represents a major public health problem because of its high rates of morbidity and mortality due to clinical complications at the chronic phase [1]. This scenario requires functional and molecular characterization of *T. cruzi* virulence factors that could serve as the basis for vaccine development or as targets for Chagas disease chemotherapy.

The pathogenesis of Chagas disease is due to the ability of *T. cruzi* to colonize, grow and persist in human tissues for years and to elicit immunopathological and parasitological reactions [2], [3]. The corresponding biological processes that account for these pathogenic mechanisms include those associated with the entry of *T. cruzi* into mammalian host cells, which involve specific interactions between host cells and many parasite proteins such as GP82, cruzipain, prolyl oligopeptidase (POP Tc80) and oligopeptidase B (OPBTc) [4]–[6]. To infect nonphagocytic mammalian cells, *T. cruzi* trypomastigotes trigger Ca^+2^-signalling in the host cells that results in the recruitment and fusion of lysosomes at the parasite binding site. Inhibition of OPBTc activity precludes entry of trypomastigote forms of the parasite into host cells. Furthermore, specific silencing of OPBTc gene greatly inhibited the infective capacity of trypomastigotes both *in vitro* and *in vivo*, revealing OPBTc as a *T. cruzi* virulence factor and, thus, a good target for developing new drugs to treat *T. cruzi* infections [7], [8].

Oligopeptidase B (OPB, EC3.4.21.83) belongs to the prolyl oligopeptidase (POP) family of serine proteases (clan SC, family S9) [9] and, unlike other POP members, does not cleave after proline residues. However, OPB shares similarities of catalytic domain amino acids and of secondary structure prediction with POP family members [10], [11]. OPB is a processing enzyme, specific for the basic amino acid pairs of peptides [12].

OPB has been described in Gram-negative and positive bacteria, spirochetes and protozoans, but not in higher eukaryotes with the exception of plants [13]-[15]. Its absence in higher animals consists of an advantage for the development of pathogen OPB inhibitors aiming at safe chemotherapy, with minimal secondary effects, to treat infected vertebrate hosts. Although significant efforts have been made towards understanding the structural and functional properties of OPB [11], [14]–[16], the physiological role of the enzyme is unknown and its natural substrate has not been identified. In addition to *T. cruzi*, OPB is increasingly being implicated as an important virulence factor of other trypanosomatids [17]. Oligopeptidase B of *Trypanosoma evansi* inactivates atrial natriuretic factor in the bloodstream of infected hosts [18]. The drugs most commonly used in sleeping sickness treatment reduce the activity of *T. brucei* oligopeptidase B [19]. Although OPB seems to be involved with regulation of parasite enolase and immune evasion in *Leishmania donovani* [20], it is not a virulence factor for *Leishmania major* [21]. OPBTc has a predicted molecular mass of nearly 80 kDa, although it migrates as an 120-kDa protein in semi-native SDS–PAGE, corresponding neither to a monomer nor to a dimer [22]. Afterward, the dimerization of *T. cruzi* oligopeptidase B was suggested through gel filtration assays but it has never been confirmed [7]. Further structural and functional characterization of OPBTc should assist in the development of specific inhibitors that will be useful for probing OPBTc physiological roles and that may have potential therapeutic applications.

In this work we show the dimeric nature of OPBTc and the structural modifications accounting for the variations of its enzymatic activity under different conditions of pH, salt concentration and temperature.

## Materials and Methods

### Parasites

*T. cruzi* epimastigote forms from CL Brener stock were grown in liver infusion tryptose medium (LIT) supplemented with 100 µg/mL of Gentamicin and 5% (v/v) fetal calf serum at 28°C [23].

### Cloning, Expression and Purification of *T. cruzi* Oligopeptidase B

Specific primers OPB1 (forward, 5′ attctaCTCGAG **ATG**AAGTGTGGTCCCATTGC 3′; lowercase, random bases; underlined, *XhoI* site; bold, initiation codon) and OPB2 (reverse, 5′ attctaGGATCC **TCA**CCTCCGAAGAAGTGTCC 3′; lower case, random bases; underlined, *BamHI* site; bold, stop codon) were designed from Oligopeptidase B gene (*opbtc)* sequence (Tc00.1047053511557.10; www.genedb.org). The *opbtc* ORF was amplified by PCR using OPB1 and OPB2 primers from genomic *T. cruzi* DNA (CL Brener). The PCR product was subsequently cloned into pGEM-T easy vector (Promega) and completely sequenced on both directions. The ORF was excised from the vector using *XhoI* and *BamHI* enzymes and subcloned into a previously *XhoI* and *BamHI*-digested pET19b plasmid (Novagen), generating pET19b/*opbtc*. Prediction of OPBTc secondary structure was performed with PSI-PRED and JPred methodology (Fig. S1).

The active OPBTc was produced in *Echerichia coli* BL21(DE3) and purified by affinity chromatography. Briefly, the N-terminal His-tagged OPBTc was expressed in *E. coli* BL21(DE3) by induction of a log phase culture with 0.5 mM isopropylthio-β-D-galactoside (IPTG) at 28°C over 16 h. Cells were harvested, lysed with Bugbuster™ (Novagen) and submitted to centrifugation at 16,000 *g* for 20 min at 4°C. The recombinant protein was purified using His-Bind Kit (Novagen). To verify the purity and yield, purified OPBTc was subjected to 10% SDS–PAGE under reducing conditions followed by Coomasie Blue staining. Protein concentration was determined using the molar absorption coefficient ε value of 118,775 (M^−1^cm^−1^) at 280 nm measured in water.

### Assay of Enzyme Activity

Recombinant OPBTc activity was determined by measuring the fluorescence of 7-amido-4-methylcoumarin (AMC) released by hydrolysis of the enzyme substrate *N*-CBZ-Gly-Gly-Arg-AMC [22]. Purified OPBTc was assayed in reaction buffer, 25 mM Tris-HCl pH 8.0, containing 20 µM substrate in 100 µL final volume at different concentrations of DTT and NaCl. AMC release was recorded up to 20 min at 355 nm excitation and 460 nm emission in a 96-well microplate fluorescence reader. The pH activity optimum of OPBTc was determined in AMT (100 mM acetic acid, 100 mM MES and 200 mM Tris-HCl) at pHs raging from 5.0 to 10.0 as previously described [24]. The temperature assay was carried out by incubating the enzyme at each temperature for 20 min and afterwards 20 µM of substrate were added to a final volume reaction of 50 µL. After 20 min, 150 µL ethanol were added to stop the reaction. In this case, AMC release was recorded as endpoint, a value of accumulated fluorescence.

### *In-gel* Proteolytic Activity

Proteolytic activity in SDS–PAGE was carried out as described [22]. Briefly, 1 µg OPBTc was solubilized in electrophoresis sample buffer with or without SDS or DTT at room temperature. After the electrophoresis at 4°C, the gel was washed twice in 2.5% Triton X-100 for 30 min, four times in 25 mM Tris-HCl pH 8.0 at 4°C and incubated for 2 min with 20 µM of *N*-CBZ-Gly-Gly-Arg-AMC at room temperature. Enzymatic activity was visualized in an ultraviolet light box.

### Analytical Ultracentrifugation

Sedimentation velocity experiments were performed using a Beckman XL-I analytical ultracentrifuge and an AN-60 TI rotor (Beckman Coulter). The experiments were carried out at 10 °C for OPBTc at 28.1, 8.4 and 1.8 µM in 25 mM Tris pH 8.0, 100 mM NaCl. A volume of 100 µL (for the most concentrated sample) or 400 µL was loaded into 0.3 or 1.2 cm path cells and centrifuged at 130,000 × *g* (42,000 rpm). Scans were recorded every 6 min, overnight, at 280 nm and by interference. We used the Sednterp software (free available at http://www.jphilo.mailway.com/) to estimate the partial specific volume of the polypeptide chain, 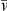  = 0.727 mL/g, the solvent density, *ρ* = 1.00458 g/mL, and the solvent viscosity, *η* = 1.3072 mPa.s, at 10 °C. Sedimentation profiles were analyzed by the size-distribution analysis of Sedfit (free available at http://www.analyticalultracentrifugation.com). In Sedfit, finite element solutions of the Lamm equation for a large number of discrete, independent species, for which a relationship between mass, sedimentation and diffusion coefficients, *s* and *D*, is assumed, are combined with a maximum entropy regularization to represent a continuous size-distribution [25]. We used 200 generated sets of data on a grid of 300 radial points, calculated using fitted frictional ratio for sedimentation coefficients comprised between 1 and 15 S. For the regularization procedure a confidence level of 0.68 was used.

### Size Exclusion Chromatography

An Akta Purifier system equipped with a Superdex 200 10/300 analytical column (GE healthcare) was used to estimate the protein apparent molecular mass in solution. Before each run, the column was equilibrated with 25 mM acetate pH 4.0; 5.0; 6.0 or Tris-HCl pH 7.0 or 8.0 containing 200 mM NaCl. Samples were diluted in the buffer used for column equilibration and 100 µL were injected and resolved at a flow rate of 0.5 mL/min at room temperature. For the temperature assays, the enzyme was incubated at 28, 37, 42, 50 or 60°C for 20 min before each run. The column was previously equilibrated with 25 mM Tris-HCl pH 8.0 containing 0.2, 0.5 or 1 M NaCl. Absorption at 280 nm was monitored and 500 µL fractions were collected and analyzed by standard PAGE in the presence of 0.2% SDS and 10 mM DTT without previous heating of the samples. Proteins of known Stokes radii (Ferritin, Aldolase, Albumin, Ovalbumin, Ribonuclease) were used for column calibration.

### Chemical denaturation

Two µg of OPBTc were incubated at room temperature in the presence of increasing concentrations of urea (up to 8.0 M). After 1.0 h incubation, samples were submitted to SDS-PAGE at 4°C followed by Coomassie Blue staining. Samples were not previously boiled and electrophoresis loading buffer contained 10 mM DTT and 0.2% SDS.

### Circular Dichroism Spectroscopy

Far-UV circular dichroism (CD) measurements were carried out on a JASCO J-810 spectropolarimeter equipped with a Peltier temperature controller and a thermostated cell holder interfaced with a thermostatic bath. Far-UV spectra were recorded in 0.1-cm path length quartz cells at a protein concentration of 0.15 mg/mL (1.72 µM) in 2.5 mM of the buffers: citric acid pH 3.0, sodium-acetate pH 4.0, sodium-acetate pH 5.0, sodium-acetate pH 6.0, N-2-Hydroxyethylpiperazine-N'-2-ethanesulfonic acid (HEPES) pH 7.0, HEPES pH 8.0, 2-(N-Cyclohexylamino)ethane sulfonic acid (CHES) pH 9.0 and 4-(Cyclohexylamino)-1-butanesulfonic acid (CABS) pH 10.0. All buffers contained 200 mM NaCl. The spectra shown in this work represent the average of four accumulated consecutives scans. Secondary structure content was estimated from the CD curves adjustments using the CDNN deconvolution software (Version 2.1, Bioinformatik.biochemtech.uni-halle.de/cdnn). Thermal denaturation assays were carried out by increasing the temperature from 20 to 75°C, allowing the temperature to stabilize for 5 min before recording each spectrum. Spectra scans were recorded at increments of 5°C. Thermal denaturation was monitored at 218 nm.

Near-UV CD spectra were acquired on a Jasco J-810 spectrophotometer with a scan speed of 50 nm.min^−1^. Spectra are the average of 15 scans and were corrected with buffer's spectrum. Data were collected from 320 to 250 nm, using 1.0×1.0-cm cells and protein concentration of 0.4 mg/mL under stirring. Buffers are the same mentioned above. For thermal denaturation experiments, temperature was controlled by a Peltier and water bath device. Thermal denaturation was monitored at 283 and 290 nm.

Ellipticity values ([θ]*_obs_*) were baseline corrected by subtracting each buffer's spectrum and converted to the mean residue ellipticity [θ]*_MRW_* in deg.cm^2^dmol^−1^, according to the equation:
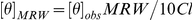
where C is the protein concentration (mg/mL), *l* is the path length (cm) and MRW is the average residue weight of OPBTc.

### Fluorescence Spectroscopy

Fluorescence measurements were performed using an ISS K-2 (Champaign, IL) spectrofluorimeter at 25°C. Spectra were recorded from 305 to 450 nm using an excitation wavelength of 295 nm and 2 nm bandwidth for both excitation and emission. Solutions of 0.87 µM OPBTc were prepared in the buffers: 2.5 mM sodium-acetate pH 5.0, sodium-acetate pH 6.0, HEPES pH 7.0, Tris-HCl pH 8.0, CHES pH 9.0. Measurements were carried out in a 1.0×1.0-cm cuvette. The final spectra were baseline corrected by subtracting each buffer's spectrum.

### ANS Fluorescence

Measurements of 1-anilino-naphthalene-8-sulfonate (ANS) fluorescence were performed as previously described [26]. Briefly, the spectra were acquired on a Jasco FP-6500 fluorimeter with the excitation wavelength set at 370 nm (5 nm slit) and emission monitored from 400 to 600 nm (5 nm slit). Five accumulations were averaged and buffer spectra were subtracted. The concentration of ANS in water was determined by measuring the optical density at 350 nm and considering a molar extinction coefficient of 4950 cm^−1^ M^−1^. Proteins were diluted to 1 µM in 25 mM HEPES pH 8.0 and were tested in a 1 cm optical path cell with 10 µM ANS. Temperature was controlled by a water-bath and measured in each sample under stirring.

## Results

### OPBTc is a Dimeric Enzyme

**Figure 1:**
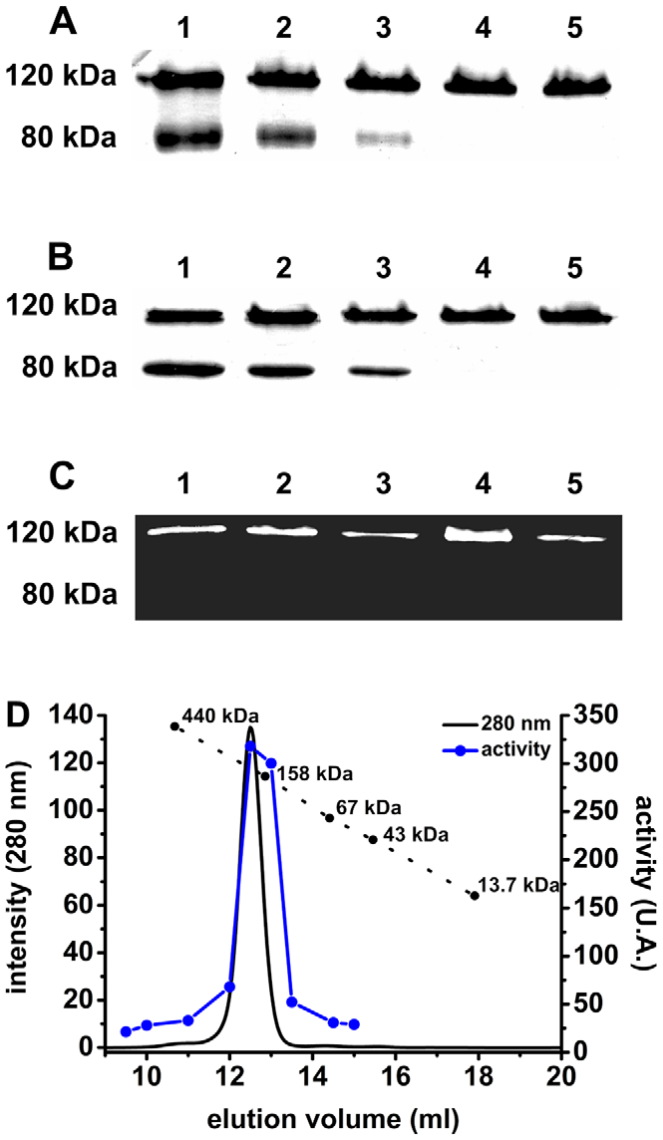
Analysis of OPBTc under SDS–PAGE and size exclusion chromatography. (A) 2 µg of OPBTc was subjected to 10% SDS–PAGE with buffer loader containing 4% (1), 1% (2), 0.5% (3), 0.1% (4) or 0.05% (5) final SDS concentration without DTT or (B) with 10 mM DTT. (C) OPBTc (500 ng) *in gel* activity was detected by incubating a replica of the gel in Fig. 1B with the fluorogenic substrate *N*-CBZ-Gly-Gly-Arg-AMC as described in Material and Methods. (D) Size Exclusion Chromatography of OPBTc was followed by activity assay at pH 8.0. The dotted black line with circles indicates the elution volumes of reference proteins. A.U. means arbitrary fluorescence units.

The active recombinant OPBTc was expressed in *E. coli* and shares similar properties with its native form purified from *T. cruzi* [7], [22], [27]. One of these features is the peculiar pattern of migration in SDS–PAGE at the apparent molecular mass of 80 kDa when fully denatured, and at 120 kDa when submitted to electrophoresis in sample buffer without SDS, DTT and prior boiling. To investigate whether the two bands corresponded to different states of OPBTc and whether SDS and DTT could interfere with that pattern, the purified enzyme was incubated with sample buffer in the presence of different concentrations of those reagents before SDS–PAGE (Fig. 1A and B). Upon staining of the gel, the revealed pattern indicated that DTT did not modify OPBTc migration pattern. In contrast, the 80-kDa band was only observed with higher concentrations of SDS in sample buffer. A single 80-kDa band was seen in the gel when the sample had been heated before electrophoresis (data not shown). These results indicate that the dimeric assembly of OPBTc does not depend on interchain disulfide bridges.

The activity of OPBTc was assayed by an *in-gel* procedure that allowed us to identify the apparent molecular mass of the active enzyme. For this, a replica of the gel in Figure 1B was washed for SDS removal and incubated with *N*-CBZ-Gly-Gly-Arg-AMC at pH 8.0. Under these conditions, a single 120-kDa fluorescent band corresponding to substrate hydrolysis was revealed (Fig. 1C). These results suggest that the band at 120-kDa corresponds to the conformation of OPBTc required for *in-gel* activity.

Since the calculated molecular mass of OPBTc is 80 kDa and the 120-kDa band corresponds neither to a monomeric nor to a dimeric state, the apparent molecular mass of the active OPBTc in solution was assessed by size exclusion chromatography in 25 mM Tris, 200 mM NaCl, pH 8.0 (Fig. 1D). Under the conditions of this experiment, a single peak was observed with an elution volume corresponding to a globular protein of 189-kDa (Stokes radius of 5.0). Enzymatic activity of each collected fraction was assayed on *N*-CBZ-Gly-Gly-Arg-AMC (Fig. 1D) and the activity peak superimposed with the single protein peak which matches with a dimer. Since both electrophoresis and size exclusion chromatography only give apparent molecular masses of the proteins, the dimeric state of OPBTc was confirmed by analytical ultracentrifugation (AUC), a versatile and powerful tool for the identification of oligomeric states and the determination of molecular masses of proteins [28].

**Figure 2:**
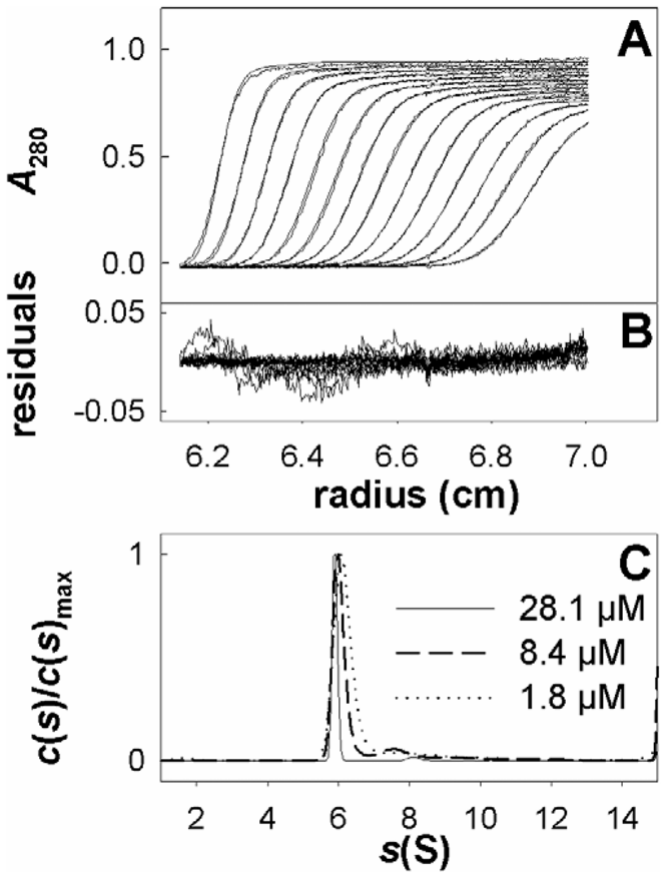
Sedimentation velocity experiments of purified *T. cruzi* oligopeptidase B. (A) Superimposition for oligopeptidase B at 28.1 µM in 25 mM Tris pH 8,0, 100 mM NaCl, of selected experimental sedimentation profiles obtained at 280 nm during 3 h at 130,000 × *g*, at 10°C, in 3 mm cell and of their modelled profiles with the *c*(*s)* analysis. (B) Corresponding residuals. (C) Result of the *c*(*s*) analysis for oligopeptidase B at 28.1, 8.4 and 1.8 µM.

Figure 2 shows the experimental and fitted sedimentation velocity profiles obtained at 28.1 µM and the superposition of the distributions *c*(*s*) of sedimentation coefficients, *s*, obtained with OPBTc at 1.8, 8.4 and 28.1 µM. The three samples show essentially the same behavior, taking into account the different signal over noise in the experimental profiles and limited non-ideal effects leading to smaller *s*-values at larger protein concentrations. A main species sediments at 6.2S (s_20,w_ = 8.2 S). The *s*-value depends on the molar mass, *M*, and Stokes radius, *R*_S_, of the particle, according to the Svedberg equation:
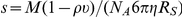



The combination of the *s*-values with *R*_S_ = 5.0 nm estimated from calibrated size exclusion chromatography gives an estimate for the OPBTc complex of *M* = 171 kDa close to the expected molecular mass for a dimer (168.4 kDa). Considering a dimer, the *R*_S_ value corresponds to a frictional ratio of 1.35, a value slightly above the usual one of 1.25 for compact globular proteins. It suggests that OPBTc dimer has an elongated or extended shape. About 10% of larger species are also detected, at about 9 S (s_20,w_ = 12S), the percentage increasing with dilution. Most probably aggregates of dimers are formed in an aging process (the fact that a larger concentration protects from aging is a commonly observed feature). These data clearly indicate that active OPBTc is an oligomeric protein formed by two monomers.

### Effects of NaCl, DTT and pH on OPBTc Catalysis

**Figure 3:**
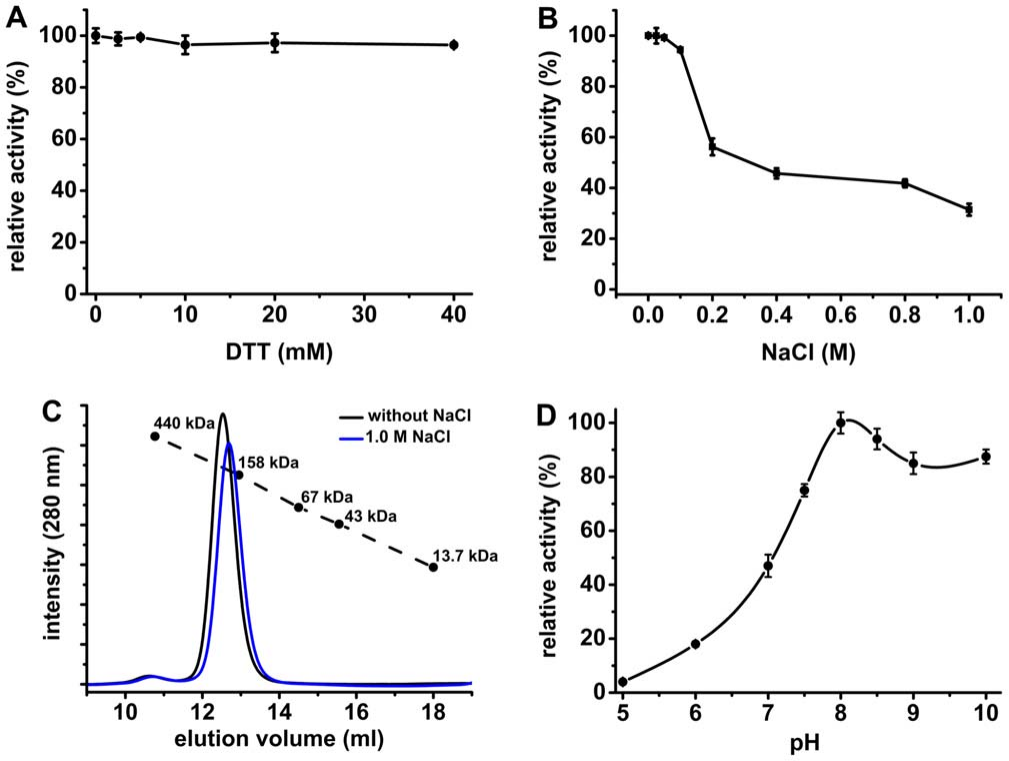
Effects of additives and pH on OPBTc catalysis. The substrate *N*-CBZ-Gly-Gly-Arg-AMC was hydrolyzed by OPBTc in the presence of (A) 0-40 mM DTT or (B) 0-1.0 M NaCl. (C) Size Exclusion chromatograms of OPBTc in the absence (black line) or in the presence of 1.0 M NaCl (blue line). (D) pH optimum activity of OPBTc. Tests were carried out as described in Material and Methods. Results are expressed as the percent activity relative to the maximum value obtained at each condition.

Two common features of the oligopeptidase B family are the improvement of its activity by thiol-reacting reagents and its sensitivity to salts [15]. Based on that, we tested the effects of these two additives on OPBTc enzymatic activity. Surprisingly, the presence of up to 40 mM DTT in the reaction buffer did not interfere with the hydrolytic activity of OPBTc on *N*-CBZ-Gly-Gly-Arg-AMC (Fig. 3A). This result correlates well with the observation that the migration pattern of OPBTc is not affected by the presence of DTT both in SDS–PAGE (Fig. 1A) and in size exclusion chromatography (data not shown). Thus, it can be concluded that interchain disulfide bonds are not essential for the stabilization of the active OPBTc dimer. To evaluate the effect of salt on the activity of the enzyme, we assayed the hydrolysis of *N*-CBZ-Gly-Gly-Arg-AMC by purified OPBTc in the presence of increasing concentrations of NaCl (Fig. 3B). Under these conditions, the activity of the enzyme showed to be very sensitive. The enzyme lost almost 50 and 70% of its activity in the presence of 0.2 and 1.0 M NaCl, respectively. To investigate whether the inhibition of the activity by NaCl was due to the induction of OPBTc monomerization, the active enzyme was subjected to size exclusion chromatography in the presence of 1.0 M NaCl (Fig. 3C). In this condition, the enzyme was eluted from the column as a single peak at an elution volume very similar to that in the absence of salt, demonstrating that NaCl did not disrupt the dimeric structure of OPBTc.

We also determined the pH-optimum profile of OPBTc using constant ionic strength buffers (Acetate, Mes and Tris). As presented in Figure 3D, the enzyme was almost inactive over the acidic pH range. At neutral pH range, the enzyme had approximately 80% of its maximal activity observed at pH 8.0, which correlates well with the OPBTc purified directly from *T. cruzi* [22]. As for DTT and NaCl, we also examined the effect of pH on OPBTc dimerization by size exclusion chromatography (Fig. S2). The column was equilibrated with buffers at each pH (4.0 – 8.0) before loading the enzyme. At pH 7.0 and 8.0, the enzyme was eluted at very similar volumes. At pH 4.0, 5.0 and 6.0, the elution of the enzyme was slightly delayed (ranging from 13.0 to 13.3 ml, compared to 12.5 ml) but it was not detected at or near the volume corresponding to an 80 kDa apparent molecular mass (14.3 ml). This increased retention may be due to minor conformational changes caused by the pH on OPBTc structure, which are not enough to induce its monomerization. Consequently, we attempted to investigate the effects of pH on the conformational changes of OPBTc employing spectroscopic methods.

### Effects of pH on OPBTc Tertiary Structure

**Figure 4:**
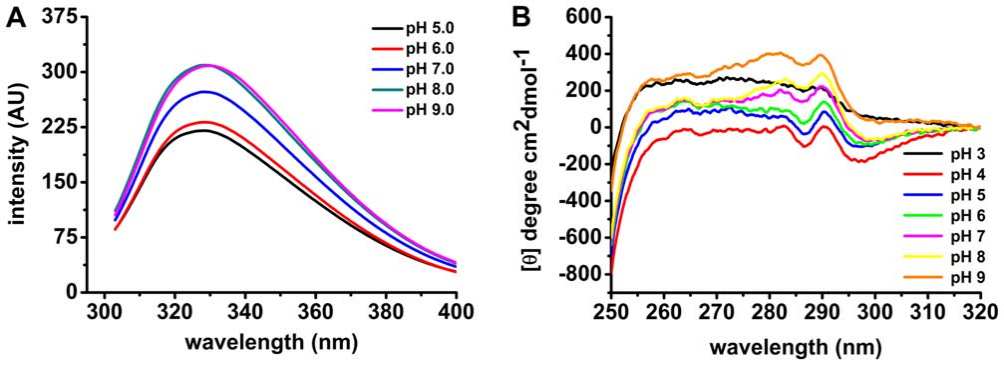
pH influence on OPBTc tertiary structure. (A) Intrinsic spectra were recorded at 25°C from 305 to 400 nm using excitation wavelength of 295 nm at different pHs. (B) Near–UV CD spectra of OPBTc at different pHs. All near–UV CD spectra were recorded at 25°C from 250 to 320 nm.

A valuable feature of intrinsic protein fluorescence is the high sensitivity of tryptophan to its local environment. Thus, it is possible to observe changes in emission spectra of tryptophan in response to protein conformational transitions, subunit association, substrate binding or denaturation, all of which can affect the local environment surrounding the tryptophan indole ring [29]. Each *T. cruzi* oligopeptidase B monomer contains 12 tryptophan residues and, as shown in Figure 4A, the emission maximum (λ_max_) of OPBTc is 330 nm, indicating that most tryptophans are partially buried in the protein. The shape of the intrinsic fluorescence spectra of OPBTc does not change from pH 5.0 to 8.0 and only a limited red-shift happens at pH 9.0. This suggests that the environment around the tryptophan residues is globally preserved over this pH range. However, it seems that at higher pHs the local environments of some of OPBTc tryptophans are apolar, leading to an increase in fluorescence intensity.

The near-UV CD spectra of proteins are mainly dependent on the relative position of each aromatic amino acid side chain. It therefore gives information, like intrinsic fluorescence, on the tertiary structure of proteins. The near-UV CD spectra of OPBTc under different pHs are presented in Figure 4B. The CD spectra displayed two peaks, one at 283 nm and the other at 290 nm, at neutral to mild-alkaline pH range (pH 7 - 9). As the pH became acidic (pH 4–6), a decay of signal at 283 nm could be observed though the signal at 290 nm did not change. Once again the changes in the enzyme tertiary structure can be well-associated with enzyme activity. The loss of signal at 283 nm, when the enzyme was incubated at mild-acidic pHs (4 - 6), indicates a conformational variation that could be responsible for OPBTc inactivation. At pH 3, the enzyme completely lost its tertiary structure (Fig. 4B) and had no activity (data not shown). We also investigated the influence of 0.2 M NaCl on OPBTc near-UV spectrum at different pHs (Fig. S3). The data showed that there was no significant difference from the spectra without NaCl. Thus far, we have demonstrated that the dimer stabilization is not due to intermolecular disulfide bonds, it is salt-resistant and OPBTc tertiary structure is sensitive to acidic pHs.

### Effects of Heat Treatment on OPBTc Dimerization and Activity

**Figure 5:**
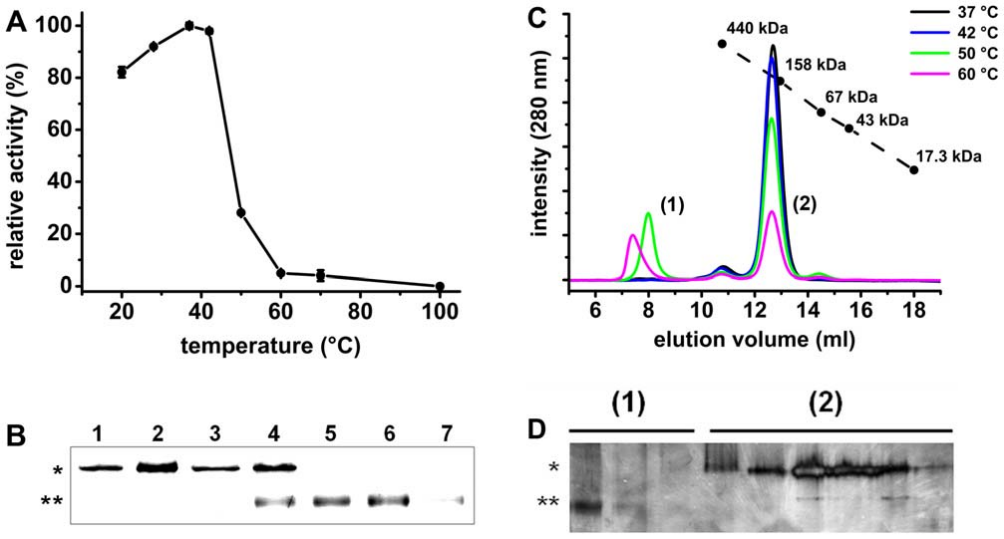
Influence of temperature on OPBTc activity and dimerization. (A) OPBTc was incubated with substrate at different temperatures for 20 min and the release of AMC was quantified as described in Material and Methods. Results are expressed as the percent activity relative to the maximum value obtained at each condition. (B) OPBTc was incubated at 28 (1), 37 (2), 42 (3), 50 (4), 60 (5), 70 (6) or 100°C (7) for 20 min in 25 mM Tris-HCl pH 8.0 followed by SDS–PAGE analysis at 4°C without previous heating; * 120 kDa; ** 80 kDa. (C) OPBTc was previously incubated at 37, 42, 50 and 60°C for 20 min and then subjected to size exclusion chromatography. (D) Fractions of OPBTc eluted from the chromatography column during the separation of the sample previously heated at 50°C. Eluted fractions were submitted to SDS–PAGE analysis without boiling the enzyme followed by silver stainning: (1) samples corresponding to the higher molecular mass (peak 1); (2) samples corresponding to the dimer (peak 2).

Thermostability studies give precious information about activity behavior and structural properties of proteins. With this purpose, we first assayed the activity of the enzyme at different temperatures (20– 100°C). At 20°C enzymatic activity corresponded to 80% of the optimal temperature activity measured when the assay was performed at 37 - 42°C, while at 50°C enzyme activity decreased drastically to nearly 25% (Fig. 5A). When the enzyme previously incubated at different temperatures was submitted to a SDS–PAGE analysis, it was observed that dimer disruption coincided with OPBTc enzymatic inactivation (Fig. 5B). Up to 42°C, OPBTc maintained its 120-kDa eletrophoretic migration pattern. In contrast, after 50°C, a transition from the 120-kDa to the 80-kDa state was clearly observed, which correlated with the temperature activity profile. Moreover, in the SDS–PAGE migration, we could also observe that the enzyme tended to aggregate at temperatures higher than 50°C. At 60 and 70°C, the 80-kDa form could be detected as well as a band with high molecular mass that did not migrate through the gel (data not shown). This aggregation could be directly related to the abrupt decrease of activity upon incubation at temperatures higher than 42°C. To address whether chemical denaturation could also promote dissociation of the dimer, OPBTc was incubated with urea prior to SDS-PAGE. Under the conditions of this experiment, the dimeric form of the enzyme was resistant to concentrations of up to 4.0 M urea. Since the monomer was progressively seen as a function of increasing concentrations of urea (Fig. S4), we concluded that urea indeed promotes disassembly of the dimeric OPBTc.

We also submitted the enzyme previously heated at the same temperatures as above to size exclusion chromatography (Fig. 5C) to assess whether the OPBTc monomer and dimer could be observed, especially at temperatures higher than 50°C when the 80-kDa form appeared in SDS–PAGE (Fig. 5B). Up to 42°C, OPBTc was eluted as a dimer. At 50 and 60°C, we observed two peaks. The first, eluting with the column void volume, corresponds to the high molecular mass aggregated enzyme. Surprisingly, the second peak was eluted as an 160-kDa protein. Once more, under these conditions, the 80-kDa form of the enzyme was not detected. However, when the high molecular mass peak was analyzed in SDS–PAGE, a single 80-kDa band was detected (Fig. 5D; lane 1). This clearly demonstrates that the 80-kDa bands observed in SDS–PAGE correspond to a denaturated state of the enzyme.

### OPBTc Structural Thermostability

**Figure 6:**
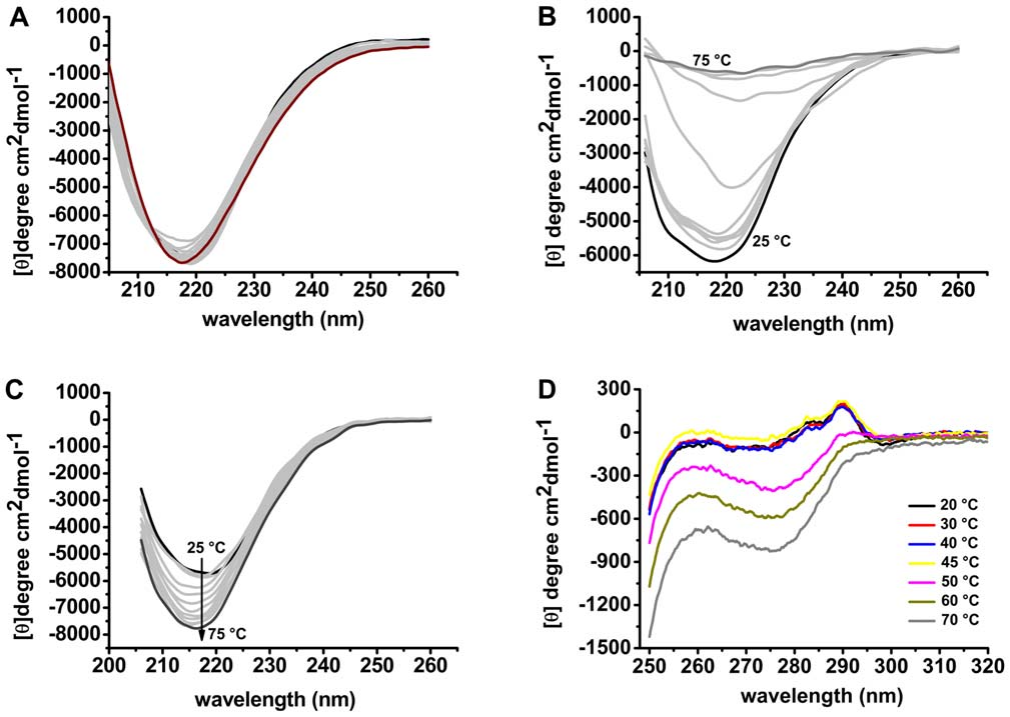
Temperature-dependent structural changes of OPBTc monitored by Far– and Near–UV CD. (A) Far–UV CD spectra at pH 8.0 at temperatures ranging from 25 (black line) to 95°C (purple line) in the absence of NaCl. (B) Far–UV CD spectra at pH 8.0 in the presence of 0.2 M NaCl at temperatures ranging from 25 (black line) to 75°C (dark gray line). (C) Far–UV CD spectra at pH 3.0 in the presence of 0.2 M NaCl. Intermediate temperatures are represented in light gray lines. (D) Near–UV CD spectra at pH 8.0 in the absence of NaCl.

The singular behavior of OPBTc after heat treatment prompted us to deepen our studies by using circular dichroism (near- and far-UV CD), which has been extensively used to give valuable information about protein structure and the extent and rate of structural changes [30]. We started our analysis by monitoring the thermal unfolding of OPBTc by far- UV CD. OPBTc spectrum at pH 8.0 and 25°C presented a characteristic band at 218 nm corresponding to β-structure (Fig. 6A). This correlates well with OPBTc secondary structure prediction, since β-strands are the major components of the β-propeller domain of the POP family [31]. Surprisingly, at pH 8.0, the protein spectra patterns were similar from 20 to 95°C (Fig. 6A). According to all the adjusted spectra, temperatures up to 95°C have no effect over the secondary structure of the enzyme. Similar results were obtained at pH 6.0 and 7.5 (Fig. S5 A-B). Thus, there are no changes on OPBTc secondary structure contents up to 95°C, although the enzyme tended to aggregate at temperatures higher than 50°C.

Considering the high thermal stability of the secondary structure of OPBTc at different pHs, as described above, we investigated the unfolding curves at mild ionic strength (0.2 M NaCl) to reduce the inter/intramolecular interactions that stabilize protein structure. Figure 6B shows far-UV CD spectra from 20 to 75°C at pH 8.0 in the presence of 0.2 M NaCl. The intensities of the dichroic bands decreased with the increase in temperature indicating complete unfolding of the protein at 75°C. Spectra pattern at pH 4.0, 7.0 and 10.0 displayed the same feature (Fig. S5 C-E). In these assays, the ionic strength promotes a perturbation of the molecular environment leading to a loss in protein secondary structure stability. However, OPBTc did not unfold at pH 3.0 with 0.2 M NaCl (Fig. 6C) and a gain in the dichroic bands was observed while temperature increased. These results strongly indicate that OPBTc has a very stable secondary structure at acidic pH. On the other hand, near-UV CD experiments at pH 3 (Fig. 4B) revealed that OPBTc lacks rigid tertiary structure at this pH. These two features and the intrinsic fluorescence λ_max_ of OPBTc at 340 nm and pH 3 (data not shown) indicate that OPBTc adopts a molten globule conformation under these conditions [32].

In order to examine the behavior of OPBTc tertiary structure upon heat treatment, we followed its unfolding process by near-UV CD. As stated before, OPBTc near-UV CD spectra at pH 8.0 and 25°C displayed two peaks, one at 283 nm and the other at 290 nm (Fig. 4B). The spectra remained stable up to 45°C, but suffered abrupt changes from 50°C to 70°C, with a notable loss of the specific signal at 283 nm and 290 nm, revealing OPBTc unfolding (Fig. 6D).

**Figure 7:**
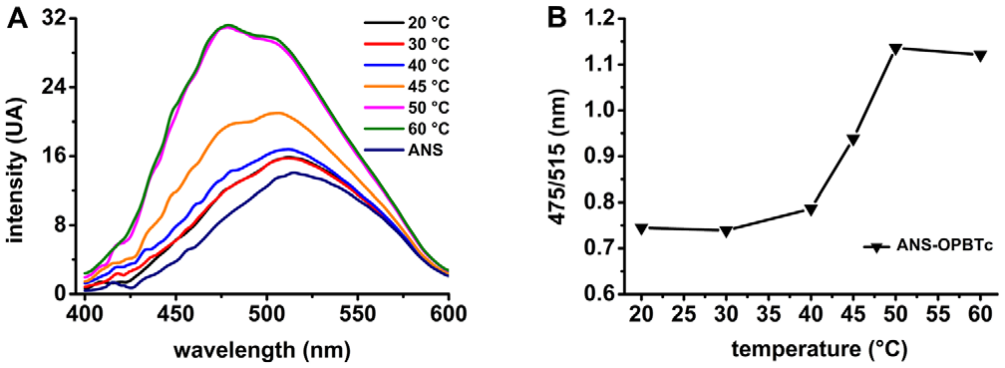
Temperature-dependent structural changes of OPBTc monitored by ANS-binding. (A) ANS-OPBTc fluorescence spectra at pH 8.0 at temperatures ranging from 20 (black line) to 60°C (green line) in the absence of NaCl. Spectrum of ANS alone is shown as a reference. (B) Ratio of ANS-OPBTc fluorescence intensities at 475 and 515 nm as a function of temperature.

This unfolding was further examined by ANS-binding studies. ANS is a worthwhile water-soluble probe for monitoring structural changes because its fluorescence changes upon binding to exposed hydrophobic clusters of proteins. OPBTc was then incubated with ANS and spectra were recorded at different temperatures (Fig. 7). At temperatures from 20 to 40°C, ANS-OPBTc spectra were very similar to the one obtained with ANS alone, exhibiting a characteristic emission peak at 515 nm. This indicates that very few hydrophobic clusters are accessible to ANS in the native enzyme. In contrast, at temperatures above 45°C, a decrease of the 515 nm peak could be observed along with the emergence of a peak at 475 nm, the mark of ANS binding (Fig. 7A). Of note, at 70°C, ANS-binding was slightly reduced most likely because of protein aggregation. These features are well illustrated when reporting the ratio of fluorescence emission intensity at 475 to 515 nm (Fig. 7B).

This temperature-induced unfolding was further confirmed by intrinsic fluorescence measurements at different temperatures, showing the exposure of tryptophan residues at temperatures above 45°C (Fig. S6). Taken together, the near-UV CD and ANS-binding studies correlate well with OPBTc activity assays: the enzyme becomes sharply inactive after 42°C due to a loss of its tertiary structure.

## Discussion

In this study, we examined some structural properties of OPBTc, a key protease for triggering Ca^2+^-signaling in both parasite and host cells, a crucial step to *T. cruzi* infection process. The oligomeric state of OPB has been a matter of question for many years. Although the crystallographic structure of *L. donovani* OPB was recently solved, this issue was not evaluated [33]. The work by Santana et al. [22] has demonstrated that the activity of native OPBTc is associated with a form much larger than 80 kDa. Morty et al. have suggested that OPB of *Trypanosoma brucei* does not form dimers, or any other kind of multimers, in the absence or presence of reducing agents, which was evident from its electrophoretic and chromatographic patterns [34]. However, as for OPBTc, the dimerization of *T. brucei* OPB has also been suggested by size exclusion chromatography [16]. While chromatography is a technique widely employed to estimate the apparent molecular mass of a protein, analytical ultracentrifugation is the method of choice for accurate molar mass determination and the study of self-association and heterogeneous interactions [28]. Therefore, the present work clearly demonstrates the dimeric nature of OPBTc. Considering that *T. cruzi* circulates in nature among more than 150 species and thousands of different cells and tissues, it certainly faces many distinct physical and chemical environments. This scenario requires that virulence factors such as OPBTc be stable enough to display functional activity under those milieu conditions. The dimeric nature of OPBTc may increase its stability and thus represent an adaptative advantage to *T. cruzi* infective forms.

None of the OPB characterized until now have presented an electrophoresis migration pattern similar to that of OPBTc, whose dimer migrates faster than expected. Despite the wide application of SDS–PAGE as a method for estimating protein *M*r, considerable uncertainty still exists about the structure of SDS–protein complexes, as well as the mechanism by which they migrate through polyacrylamide gels [35]. The migration pattern of OPBTc is intriguing since the 120-kDa band that appears in SDS–PAGE corresponds neither to its monomer nor to its dimer. In practice, for most proteins at a single gel concentration, differences in relative mobility are a function of molecular size and shape rather than charge. Anomalous migration of proteins in SDS–PAGE, for which *M*r would be incorrectly estimated, might be due to an uncommon charge to friction ratio, e.g., as a result of glycosylation or high intrinsic charge [35]. The intrinsic charge of OPBTc could be one of the reasons for its abnormal migration in SDS–PAGE, since the molecular model of *Leishmania amazonensis* OPB (OPBLa) presented an enzyme with high negative surface [11]. If OPBTc also presents a high negative charge, it could favor its migration through SDS–PAGE, justifying the 120-kDa band instead of the predicted 160 kDa. An alternative explanation for this result would be that the OPBTc dimer is partially resistant to SDS unfolding. In this case, the partially unfolded dimer would be more compact than the random-coil counterpart, thus migrating faster. This phenomenon is usually observed with proteins harboring intra-chain disulfide bonds that migrate faster in the absence than in the presence of reducing conditions [36].

In principle, the folding of dimeric proteins can occur via many different mechanisms, ranging from the simplest mechanism of a two-state transition involving only native dimers and unfolded monomers, to also forming various numbers of monomeric or dimeric intermediates. But exactly what intermediates will be observed depends on the relative stabilities of the dimer, the dimeric and monomeric intermediates, as well as protein concentration [37]. The oligomer stability is governed to a great extent by biochemical (interface hydrophobicity, polarity, hydrogen bonds) and geometrical properties (interface size, shape, atomic packing) of its protein-protein interfaces [38]. OPBTc analytical ultracentrifugation did not detect a monomeric intermediate at any of the studied concentrations. Size exclusion chromatography under several concentrations of DTT or NaCl and different temperatures and pHs did not allow observing the monomer either. Only in SDS–PAGE, at certain conditions, the monomer appeared. Taking all those results together, OPBTc dimer interface appears to be quite stable. Since we have failed to detect any activity co-migrating with the 80-kDa form of both recombinant and native OPBTc, either upon *in-gel* activity or size exclusion chromatography assays, a further characterization of the dimer interface interaction and its dissociation is needed to state whether the monomer could mediate enzymatic activity.

Enzyme catalytic activity did not change in the presence of elevated concentrations of DTT, although a common enzymatic characteristic of the S9 family is the sensitivity of its activity to thiol-reacting reagents [15]. Among 14 cysteine residues analyzed, the residue Cys^256^ of *T. brucei* OPB was identified as one of the key amino acids that could interact with reducing reagents leading to a conformational change of the enzyme [34]. OPBTc does not have this residue in its primary sequence, what could explain its insensitivity to DTT. Yet, *S. griseus* oligopeptidase B catalysis is activated by reducing reagents independently of its single cysteine residue [15].

Intrinsic fluorescence and near-UV CD assays revealed that OPBTc tertiary structure changes as a function of pH. At pH 8.0, the optimum pH, enzyme intrinsic fluorescence was one of the highest and it decayed as the environment became acidic, where the enzyme is almost inactive. Moreover, near-UV CD spectra also correlate with enzyme activity at different pHs. The loss of the specific signal at 283 nm at acidic pHs can be interpreted as a conformational change that causes enzyme inactivation. Those results might explain why the enzyme undergoes longer retention in gel filtration at acidic pHs. As suggested by the AUC, the native dimer is rather elongated but in acidic pH conditions it could become more globular delaying its elution, or else, the enzyme is somehow denatured in such conditions leading to an abnormal behavior in gel filtration, for example by interacting nonspecifically with the matrix.

At temperatures above 50°C, OPBTc activity sharply decays from almost 100% to less than 30%. CD spectroscopy, as well as ANS-binding and intrinsic fluorescence, showed that above 50°C OPBTc tertiary structure is drastically destabilized. Even though the secondary structure appears to be highly thermostable at low ionic strength, the changes in near-UV CD spectra are clear evidence that the rigid tertiary structure is lost upon heating. Furthermore, ANS-binding and intrinsic fluorescence indicate that hydrophobic clusters become significantly exposed to the solvent at temperatures above 50°C. However, we could not detect monomerization of OPBTc at these temperatures but rather protein aggregation.

Deciphering the relation between enzymatic activity and structural features of OPBTc is a further step toward the comprehension of its role in *T. cruzi* infection process. In addition, the information gathered in this study can be of help for the identification and characterization of inhibitors, which would be of tremendous interest for research and therapeutic application. Moreover, more work will be necessary for the determination of the three-dimensional structure of OPBTc that would facilitate the comprehension of its dimeric form besides being a crucial step in the rational design of novel inhibitors.

## Supporting Information

Figure S1 **The amino acid sequence of** ***T. cruzi*** **OPB with the assigned secondary structure predicted by PSI-PRED and JPred**. β-strands are represented in blue and α-helices in orange. The catalytic triad residues are highlighted in yellow. The cysteine residues are represented in red.
(TIF)

Figure S2 **Size exclusion chromatography of OPBTc under different pH conditions.** Purified OPBTc was previously incubated at different pHs and then subjected to size exclusion chromatography.
(TIF)

Figure S3 **Influence of 0.2 M NaCl on OPBTc near–UV spectrum at different pHs.** All near–UV CD spectra were recorded at 25°C from 250 to 320 nm.
(TIF)

Figure S4 **OPBTc chemical denaturation in the presence of Urea.** Two µg of OPBTc were incubated with increasing concentrations of urea in 20 µL of Tris 25 mM pH 8.0. After 1 h incubation, samples were submitted to SDS-PAGE at 4°C followed by Coomassie Blue staining. 1 – no Urea; 2 – 1 M; 3 – 2 M; 4 – 3 M; 5 - 4 M; 6 – 5 M; 7 – 6 M; 8 – 7 M; 9 – 8 M Urea.
(TIF)

Figure S5 **Temperature-dependent structural changes of OPBTc monitored by Far**–**UV.** Far–UV CD spectra at pH 6.0 (A) and pH 7.0 (B) at 25 (black line) and 75°C (gray line) in the absence of NaCl. (C, D, E) Far–UV CD spectra at pH 4.0, 7.0 and 10.0, respectively, in the presence of 0.2 M NaCl at 25 (black line) and 75°C (dark gray line). Intermediate temperatures are represented in gray lines.
(TIF)

Figure S6 **Temperature influence on OPBTc tertiary structure.** The intrinsic spectra were recorded at pH 8.0 using excitation wavelength of 295 nm at different temperatures ranging from 20 to 70°C.
(TIF)

## References

[1] Lauria-Pires L, Braga MS, Vexenat AC, Nitz N, Simoes-Barbosa A (2000). Progressive chronic Chagas heart disease ten years after treatment with anti-*Trypanosoma cruzi* nitroderivatives.. Am J Trop Med Hyg.

[2] Cunha-Neto E, Teixeira PC, Fonseca SG, Bilate AM, Kalil J (2011). Myocardial gene and protein expression profiles after autoimmune injury in Chagas' disease cardiomyopathy.. Autoimmun Rev.

[3] Bilate AM, Cunha-Neto E (2008). Chagas disease cardiomyopathy: current concepts of an old disease.. Rev Inst Med Trop Sao Paulo.

[4] Yoshida N, Cortez M (2008). *Trypanosoma cruzi*: parasite and host cell signaling during the invasion process.. Subcell Biochem.

[5] Bastos IM, Grellier P, Martins NF, Cadavid-Restrepo G, de Souza-Ault MR (2005). Molecular, functional and structural properties of the prolyl oligopeptidase of *Trypanosoma cruzi* (POP Tc80), which is required for parasite entry into mammalian cells.. Biochem J.

[6] Fernandes LC, Bastos IM, Lauria-Pires L, Rosa AC, Teixeira AR (2005). Specific human antibodies do not inhibit *Trypanosoma cruzi* oligopeptidase B and cathepsin B, and immunoglobulin G enhances the activity of trypomastigote-secreted oligopeptidase B.. Microbes Infect.

[7] Burleigh BA, Caler EV, Webster P, Andrews NW (1997). A cytosolic serine endopeptidase from *Trypanosoma cruzi* is required for the generation of Ca2+ signaling in mammalian cells.. J Cell Biol.

[8] Caler EV, Vaena de Avalos S, Haynes PA, Andrews NW, Burleigh BA (1998). Oligopeptidase B-dependent signaling mediates host cell invasion by *Trypanosoma cruzi*.. EMBO J.

[9] Rawlings ND, Morton FR, Kok CY, Kong J, Barrett AJ (2008). MEROPS: the peptidase database.. Nucleic Acids Res.

[10] Gerczei T, Keseru GM, Naray-Szabo G (2000). Construction of a 3D model of oligopeptidase B, a potential processing enzyme in prokaryotes.. J Mol Graph Model.

[11] de Matos Guedes HL, Duarte Carneiro MP, de Oliveira Gomes DC, Rossi-Bergmann B, Giovanni De-Simone S (2007). Oligopeptidase B from *Leishmania amazonensis*: molecular cloning, gene expression analysis and molecular model.. Parasitol Res.

[12] Polgar L (1997). A potential processing enzyme in prokaryotes: oligopeptidase B, a new type of serine peptidase.. Proteins.

[13] Tsuji A, Yuasa K, Matsuda Y (2004). Identification of oligopeptidase B in higher plants. Purification and characterization of oligopeptidase B from quiescent wheat embryo, *Triticum aestivum*.. J Biochem.

[14] Rea D, Hazell C, Andrews NW, Morty RE, Fulop V (2006). Expression, purification and preliminary crystallographic analysis of oligopeptidase B from *Trypanosoma brucei*.. Acta Crystallogr Sect F Struct Biol Cryst Commun.

[15] Usuki H, Uesugi Y, Iwabuchi M, Hatanaka T (2009). Activation of oligopeptidase B from *Streptomyces griseus* by thiol-reacting reagents is independent of the single reactive cysteine residue.. Biochim Biophys Acta.

[16] Mohd Ismail NI, Yuasa T, Yuasa K, Nambu Y, Nisimoto M (2010). A critical role for highly conserved Glu(610) residue of oligopeptidase B from *Trypanosoma brucei* in thermal stability.. J Biochem.

[17] Coetzer TH, Goldring JP, Huson LE (2008). Oligopeptidase B: a processing peptidase involved in pathogenesis.. Biochimie.

[18] Morty RE, Pelle R, Vadasz I, Uzcanga GL, Seeger W (2005). Oligopeptidase B from *Trypanosoma evansi*. A parasite peptidase that inactivates atrial natriuretic factor in the bloodstream of infected hosts.. J Biol Chem.

[19] Morty RE, Troeberg L, Pike RN, Jones R, Nickel P (1998). A trypanosome oligopeptidase as a target for the trypanocidal agents pentamidine, diminazene and suramin.. FEBS Lett.

[20] Swenerton RK, Zhang S, Sajid M, Medzihradszky KF, Craik CS (2011). The oligopeptidase B of *Leishmania* regulates parasite enolase and immune evasion.. J Biol Chem.

[21] Munday JC, McLuskey K, Brown E, Coombs GH, Mottram JC (2011). Oligopeptidase B deficient mutants of *Leishmania major*.. Mol Biochem Parasitol.

[22] Santana JM, Grellier P, Rodier MH, Schrevel J, Teixeira A (1992). Purification and characterization of a new 120 kDa alkaline proteinase of *Trypanosoma cruzi*.. Biochem Biophys Res Commun.

[23] Camargo EP (1964). Growth and Differentiation in *Trypanosoma Cruzi*. I. Origin of Metacyclic Trypanosomes in Liquid Media.. Rev Inst Med Trop Sao Paulo.

[24] Bastos IM, Motta FN, Charneau S, Santana JM, Dubost L (2010). Prolyl oligopeptidase of *Trypanosoma brucei* hydrolyzes native collagen, peptide hormones and is active in the plasma of infected mice.. Microbes Infect.

[25] Schuck P (2000). Size-distribution analysis of macromolecules by sedimentation velocity ultracentrifugation and lamm equation modeling.. Biophys J.

[26] Faudry E, Job V, Dessen A, Attree I, Forge V (2007). Type III secretion system translocator has a molten globule conformation both in its free and chaperone-bound forms.. FEBS J.

[27] Burleigh BA, Andrews NW (1995). A 120-kDa alkaline peptidase from *Trypanosoma cruzi* is involved in the generation of a novel Ca(2+)-signaling factor for mammalian cells.. J Biol Chem.

[28] Lebowitz J, Lewis MS, Schuck P (2002). Modern analytical ultracentrifugation in protein science: a tutorial review.. Protein Sci.

[29] Lakowicz JR (2004). Principle of fluorescence spectroscopy..

[30] Kelly SM, Jess TJ, Price NC (2005). How to study proteins by circular dichroism.. Biochim Biophys Acta.

[31] Fulop V, Bocskei Z, Polgar L (1998). Prolyl oligopeptidase: an unusual beta-propeller domain regulates proteolysis.. Cell.

[32] Qureshi SH, Moza B, Yadav S, Ahmad F (2003). Conformational and thermodynamic characterization of the molten globule state occurring during unfolding of cytochromes-c by weak salt denaturants.. Biochemistry.

[33] McLuskey K, Paterson NG, Bland ND, Isaacs NW, Mottram JC (2010). Crystal structure of *Leishmania major* oligopeptidase B gives insight into the enzymatic properties of a trypanosomatid virulence factor.. J Biol Chem.

[34] Morty RE, Shih AY, Fulop V, Andrews NW (2005). Identification of the reactive cysteine residues in oligopeptidase B from *Trypanosoma brucei*.. FEBS Lett.

[35] Westerhuis WH, Sturgis JN, Niederman RA (2000). Reevaluation of the electrophoretic migration behavior of soluble globular proteins in the native and detergent-denatured states in polyacrylamide gels.. Anal Biochem.

[36] Braakman I, Hoover-Litty H, Wagner KR, Helenius A (1991). Folding of influenza hemagglutinin in the endoplasmic reticulum.. J Cell Biol.

[37] Rumfeldt JA, Galvagnion C, Vassall KA, Meiering EM (2008). Conformational stability and folding mechanisms of dimeric proteins.. Prog Biophys Mol Biol.

[38] Ponstingl H, Kabir T, Gorse D, Thornton JM (2005). Morphological aspects of oligomeric protein structures.. Prog Biophys Mol Biol.

