# Supplementary figures and images for: The Trypanosoma cruzi Virulence Factor Oligopeptidase B (OPBTc) Assembles into an Active and Stable Dimer
Source: PLoS One. 2012 Jan 19;7(1):e30431. doi: 10.1371/journal.pone.0030431 (PMC3261901; doi:10.1371/journal.pone.0030431)

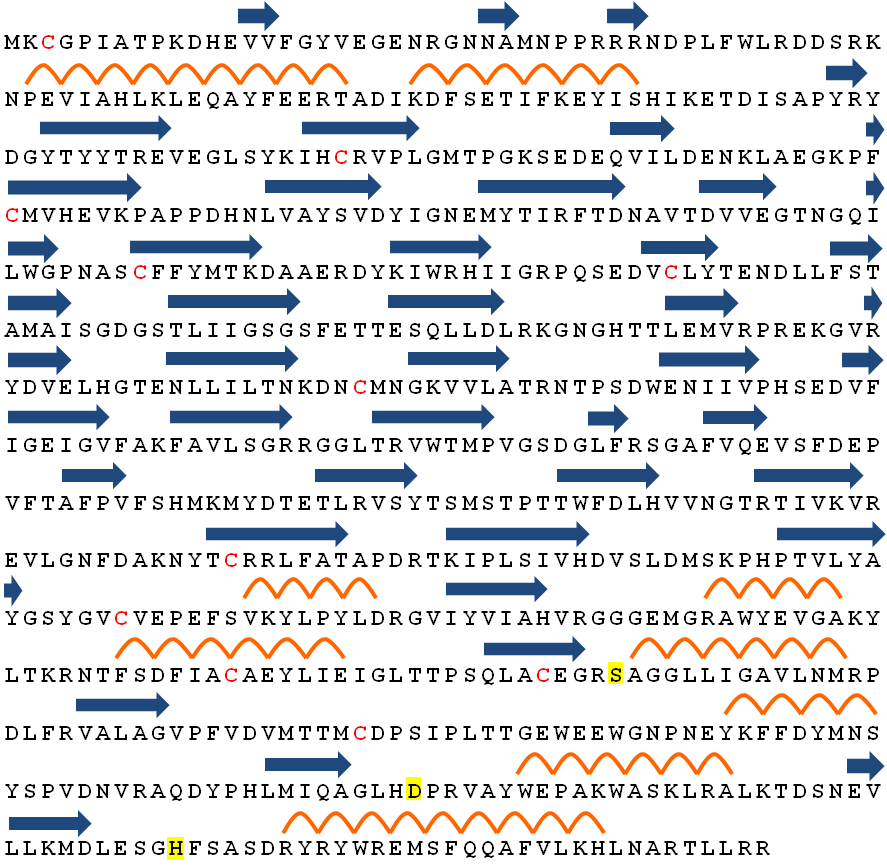

Supplement: Figure S1 — The amino acid sequence of T. cruzi OPB with the assigned secondary structure predicted by PSI-PRED and JPred. β-strands are represented in blue and α-helices in orange. The catalytic triad residues are highlighted in yellow. The cysteine residues are represented in red. (TIF) [file pone.0030431.s001.tif]

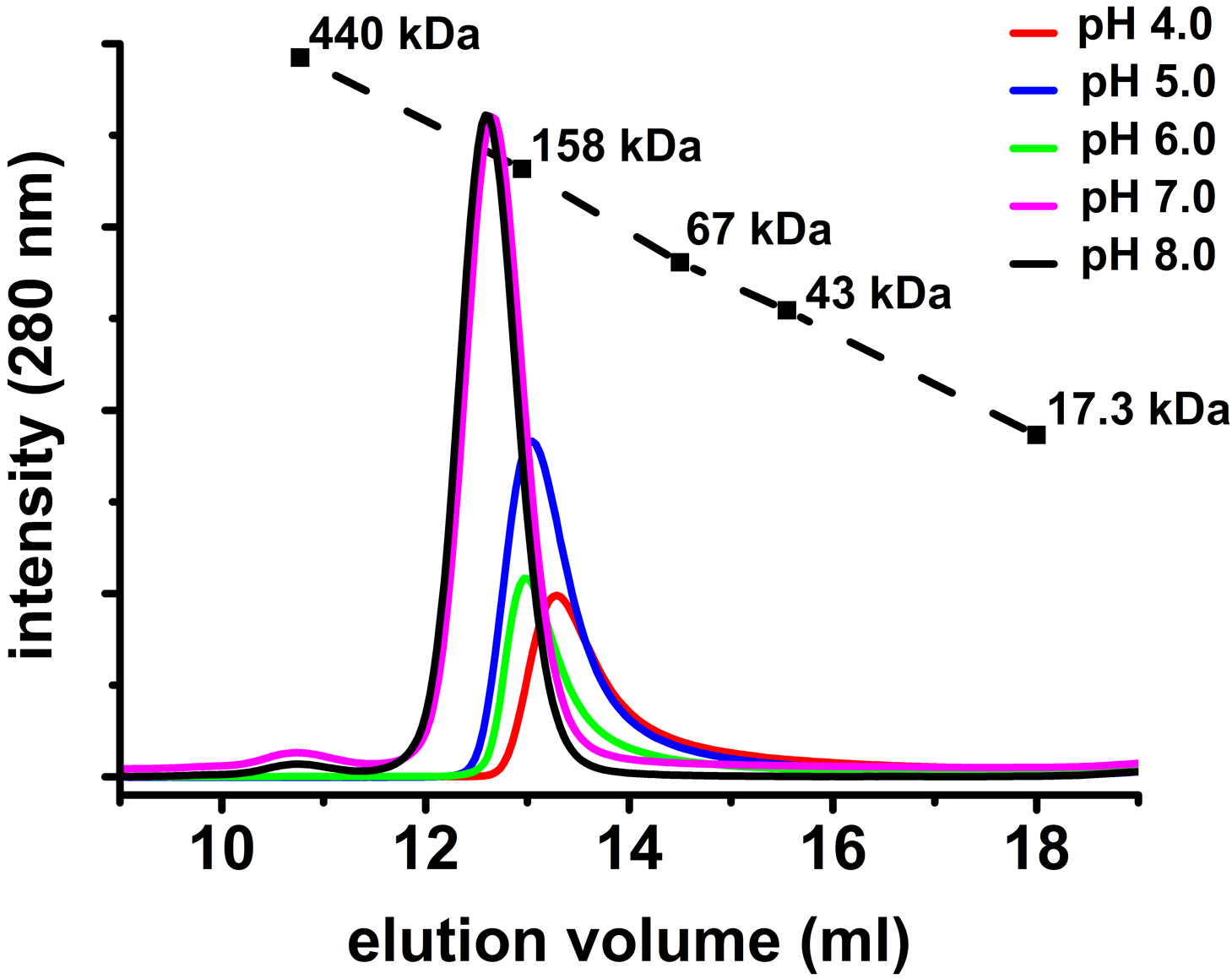

Supplement: Figure S2 — Size exclusion chromatography of OPBTc under different pH conditions. Purified OPBTc was previously incubated at different pHs and then subjected to size exclusion chromatography. (TIF) [file pone.0030431.s002.tif]

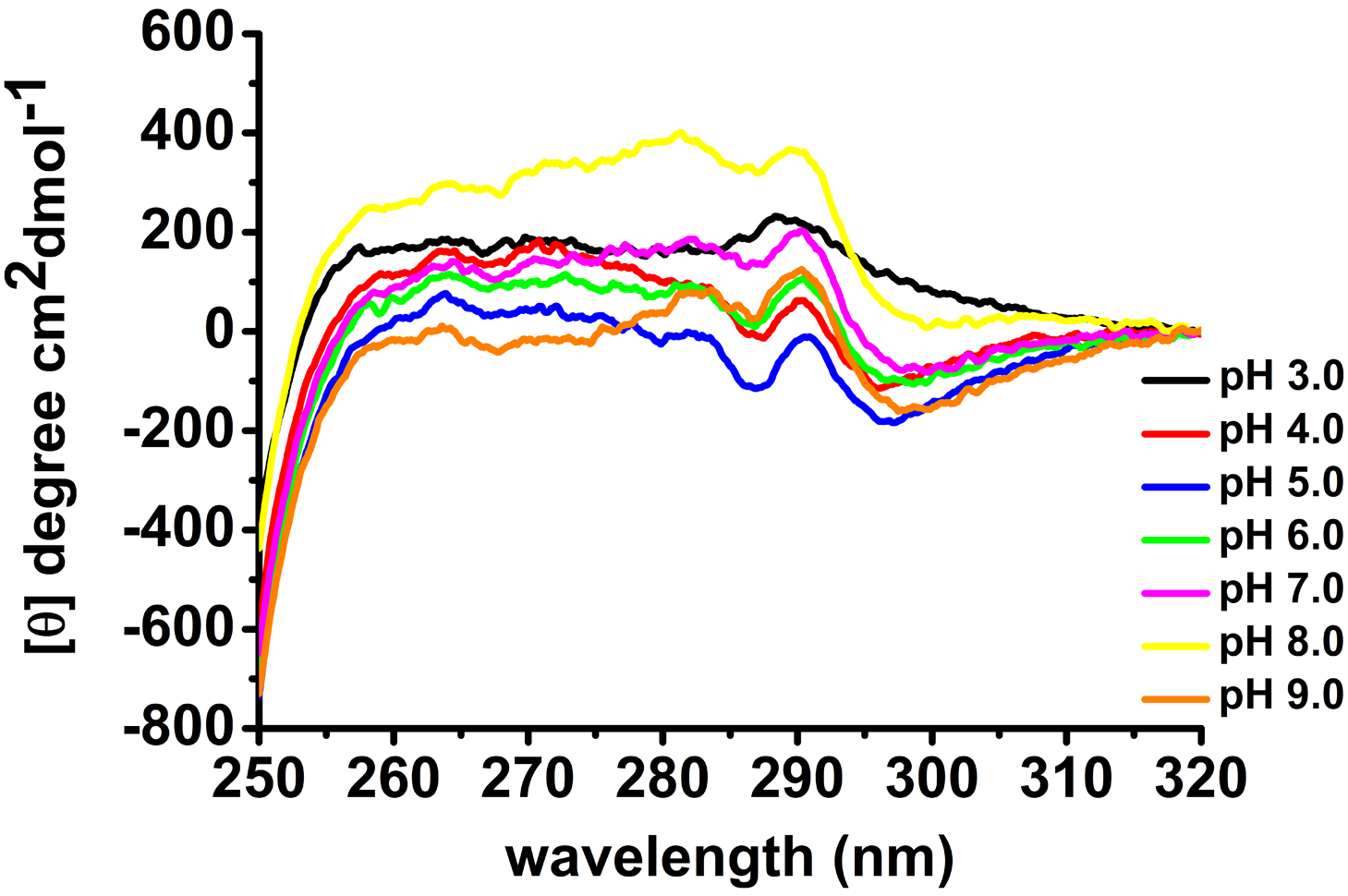

Supplement: Figure S3 — Influence of 0.2 M NaCl on OPBTc near–UV spectrum at different pHs. All near–UV CD spectra were recorded at 25°C from 250 to 320 nm. (TIF) [file pone.0030431.s003.tif]

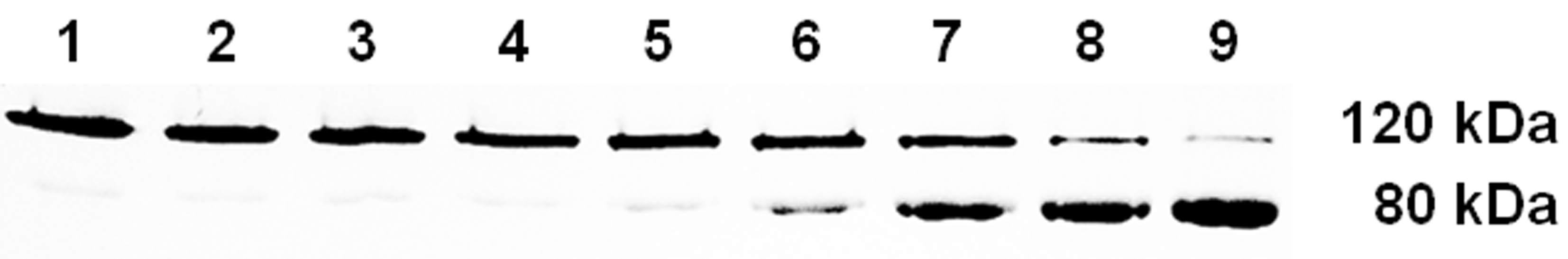

Supplement: Figure S4 — OPBTc chemical denaturation in the presence of Urea. Two µg of OPBTc were incubated with increasing concentrations of urea in 20 µL of Tris 25 mM pH 8.0. After 1 h incubation, samples were submitted to SDS-PAGE at 4°C followed by Coomassie Blue staining. 1 – no Urea; 2 – 1 M; 3 – 2 M; 4 – 3 M; 5 - 4 M; 6 – 5 M; 7 – 6 M; 8 – 7 M; 9 – 8 M Urea. (TIF) [file pone.0030431.s004.tif]

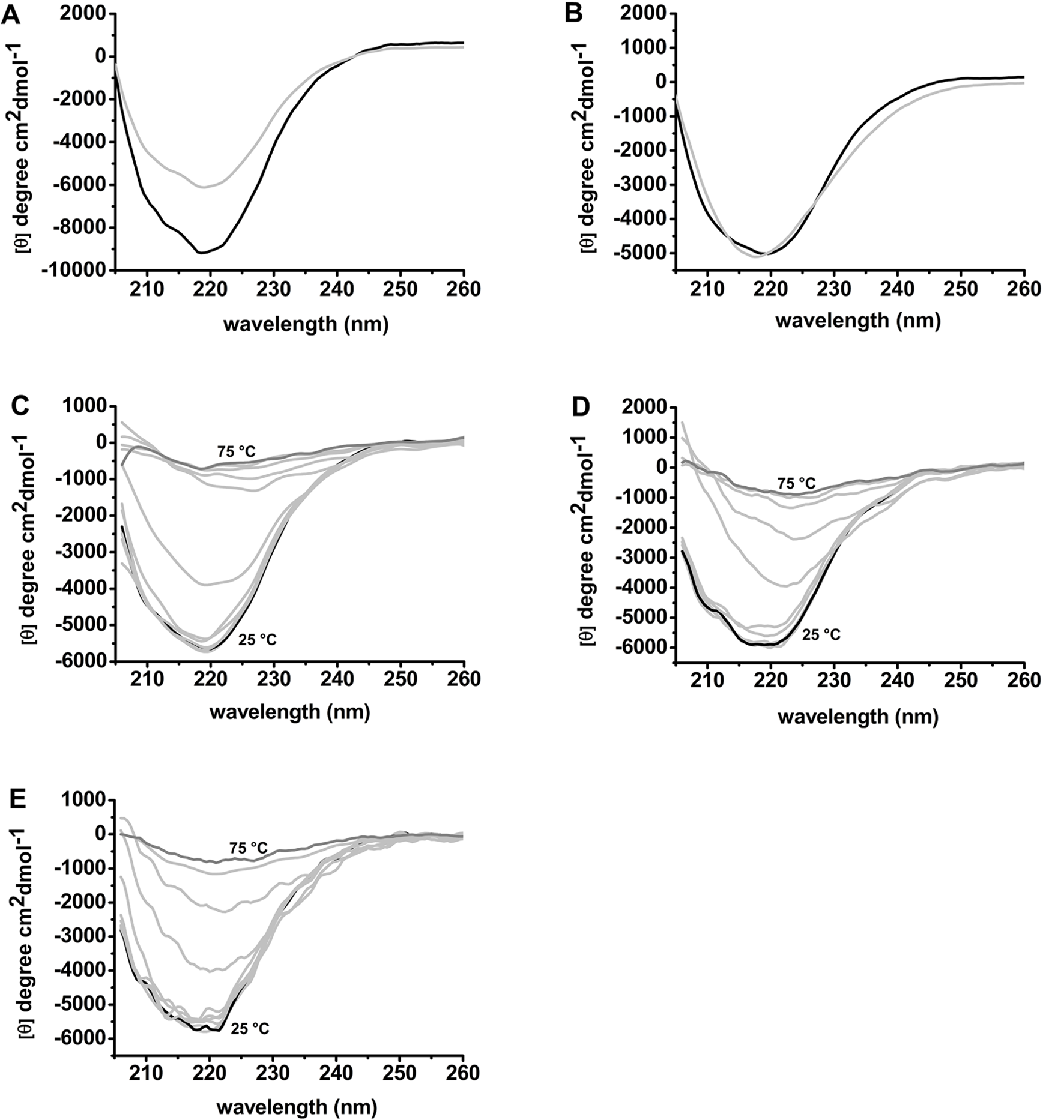

Supplement: Figure S5 — Temperature-dependent structural changes of OPBTc monitored by Far–UV. Far–UV CD spectra at pH 6.0 (A) and pH 7.0 (B) at 25 (black line) and 75°C (gray line) in the absence of NaCl. (C, D, E) Far–UV CD spectra at pH 4.0, 7.0 and 10.0, respectively, in the presence of 0.2 M NaCl at 25 (black line) and 75°C (dark gray line). Intermediate temperatures are represented in gray lines. (TIF) [file pone.0030431.s005.tif]

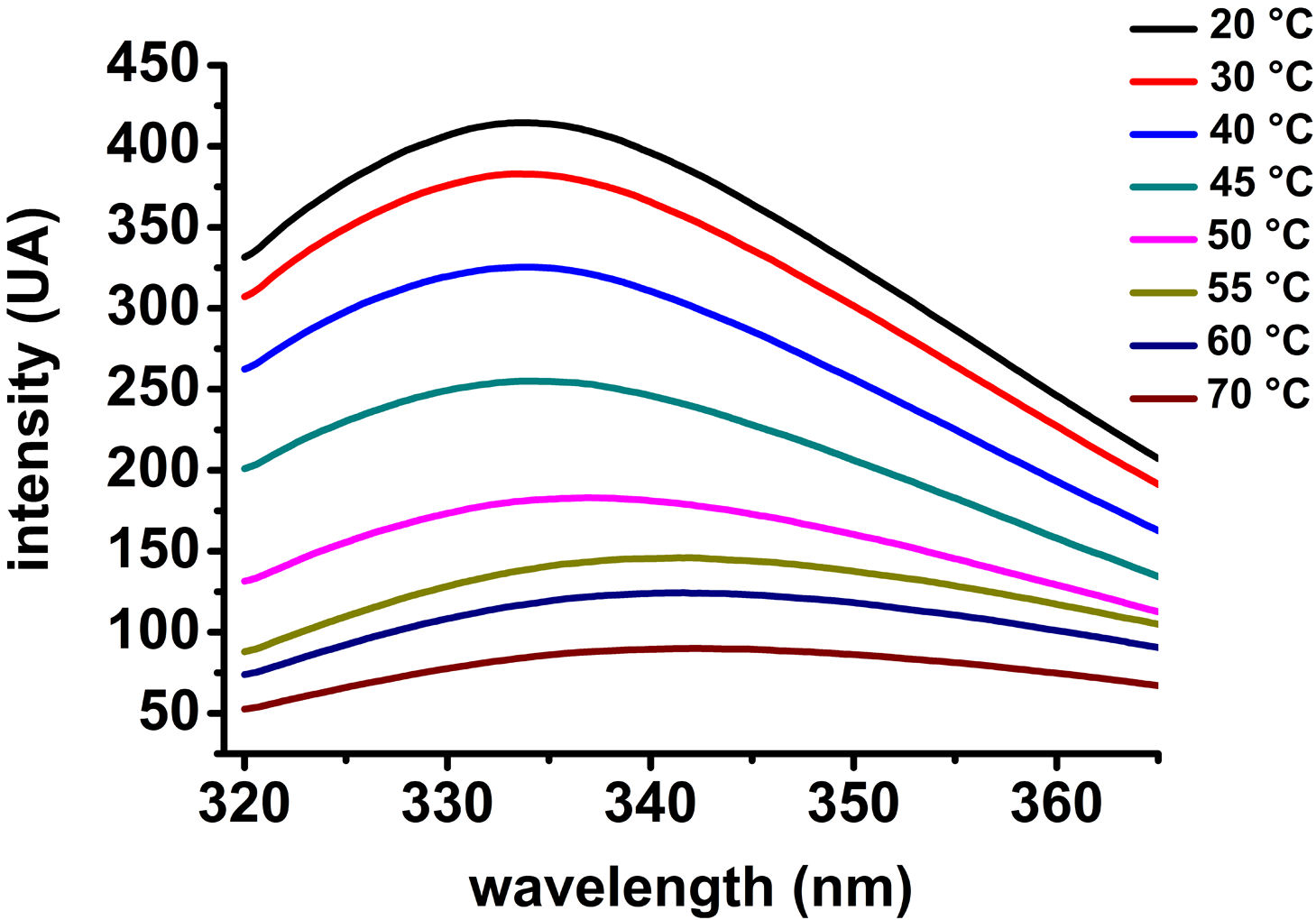

Supplement: Figure S6 — Temperature influence on OPBTc tertiary structure. The intrinsic spectra were recorded at pH 8.0 using excitation wavelength of 295 nm at different temperatures ranging from 20 to 70°C. (TIF) [file pone.0030431.s006.tif]
